# Disability inclusion assessment in primary healthcare centers in Eastern Saudi Arabia: a way forward

**DOI:** 10.25122/jml-2023-0385

**Published:** 2023-12

**Authors:** Lamees Yousef, Norah Almatroodi, Dannah AlAngari, Rahaf AlShehri, Shahad Alshammari

**Affiliations:** 1 Model of Care Department, Health Holding Company, Khobar, Saudi Arabia; 2 College of Public Health, Imam Abdulrahman Bin Faisal University, Dammam, Saudi Arabia

**Keywords:** inclusion, Saudi Arabia, disability

## Abstract

Individuals with disabilities often experience barriers in accessing healthcare facilities, including physical barriers such as inaccessible facilities, transportation difficulties, and a lack of assistive devices. Other barriers may include a lack of communication assistive devices and stigma or negative attitudes from healthcare personnel or society. Published literature emphasizes the value of creating a supportive and safe environment for the inclusion of persons with disabilities in society. Establishing guidelines for disability inclusion becomes imperative to ensure equitable access to healthcare services. This study aimed to identify challenges related to accessibility in infrastructure, services, equipment, processes, and training in primary healthcare settings. The study was conducted in Dammam, AlKhobar, and AlQatif in August 2022, using an analytical, quantitative cross-sectional approach. A total of 56 primary healthcare centers (PHCs) were assessed across multiple domains. Government-built PHCs had an average accessibility rate of 83.2%, while those located in rented buildings scored an average of 67.1%. One domain that scored highly among both building types was the clinic rooms domain, with an average score of 90%. Conversely, the services domain showed significant differences, with an average accessibility rate of 47% for rented buildings and 75% for government-built buildings. Finally, the study looked into recommendations drawn from other health systems and recommended ways to help improve disability needs inclusion in the Eastern Region of Saudi Arabia.

## INTRODUCTION

The Centers for Disease Control and Prevention (CDC) defines disability as a physical or mental impairment that imposes limitations on an individual and restricts their engagement in activities and interaction with their environment [1]. According to the World Health Organization (WHO), there are approximately one billion people worldwide living with disabilities, and this number is projected to increase. This increase can be partly attributed to the aging population and the growing incidence of non-communicable diseases [2]. Disability may refer to cognitive, hearing, visual, learning, autism spectrum disorders, and other disabilities [1].

According to data from the Authority of People with Disabilities, individuals with documented disabilities make up approximately 7.1% of the total population in Saudi Arabia [3]. King Salman Center for Disability Research in Saudi Arabia defines disability as follows: “Individuals who are totally or partially disabled with respect to their physical, sensory, mental, communicative, academic, or psychological capabilities, to the extent that it compromises the abilities to meet their normal needs as compared to individuals without disabilities” [4].

In 2018, the prevalence of disability in the Eastern Region of Saudi Arabia was 3,506 per 100,000, which is above the national average of 3,326 per 100,000 [5]. A study conducted by Bindawas and Vennu found that the disability rate in Saudi Arabia increased with age and was more prominent in men than women [5]. Additionally, the elderly population (65+) experience increased difficulties in movement, vision, and hearing due to health conditions and chronic illnesses such as diabetes and hypertension [6, 7].

Persons with disabilities often experience barriers in accessing healthcare facilities, including physical barriers such as inaccessible facilities, transportation difficulties, and a lack of assistive devices. Other barriers may include a lack of communication assistive devices and stigma or negative attitudes from healthcare personnel or society [8, 9]. A survey conducted in one of the medical cities in Saudi Arabia to assess the access of persons with mobility disabilities found that 91% of those surveyed needed help from others to reach the facilities [10]. Another study found that persons with physical disabilities had low satisfaction rates (65.8%) due to not having an emergency call button in the restrooms. The study also found that 67% of the participants were dissatisfied with the adequacy and quality of wheelchairs [11]. Published literature emphasizes the value of creating a supportive and safe environment for the inclusion of persons with disabilities in society. Therefore, it is necessary to have disability inclusion guidelines that create a standard for equitable access to care [2]. The primary objective of our study was to identify accessibility challenges within primary healthcare settings, including infrastructure, services, equipment, processes, and training. Additionally, our study sought to provide recommendations to enhance disability inclusion in the Eastern Region of Saudi Arabia.

## MATERIAL AND METHODS

### Study design and data collection

This study was conducted in August 2022 and followed an analytical, quantitative, and cross-sectional approach. We aimed to assess accessibility challenges in primary healthcare settings across the Eastern Region of Saudi Arabia. To achieve a comprehensive representation, we utilized a total population sampling technique, including all primary healthcare centers (PHCs) within Dammam, AlKhobar, and AlQatif, along with their respective networks, totaling 56 PHCs. PHCs that were temporarily or permanently closed and those outside the jurisdiction of the selected networks were excluded from the study.

### Data collection instrument

Data was collected using a validated questionnaire comprising eight main domains [12]. Each domain was designed to assess different aspects of accessibility for persons with disabilities within PHCs. The first domain was “Accessible parking,” which assessed the availability and accessibility of allocated parking spaces within PHCs. The second domain was “Accessible entrance,” which measured the infrastructure preparedness for persons with disabilities to access the building, such as the inclusion of ramps. The third domain, “Accessible services,” evaluated the convenience, space, reach, and use of assistive technology. The fourth domain, “Toilet rooms,” focused on the suitability of toilet rooms and attached equipment for persons with disabilities. The fifth domain, "Waiting rooms," measured the ease of finding the path to the waiting room and how well it accommodates persons with disabilities. The sixth domain, "Clinic rooms," assessed the accessibility of the clinic rooms with a specific focus on sufficient space for movement within doorways and rooms. The seventh domain, "Equipment," evaluated the availability of proper equipment to facilitate the treatment of persons with disabilities. Finally, the eighth domain, "Process and training” investigated healthcare providers’ knowledge and previous training to properly care for persons with disabilities (Table 1).

**Table 1:** Description of assessment domains

Domain	Description
Parking	Evaluates the accessibility of the parking area, including the availability of designated parking spots, their distance from the building, and the width of parking spaces.
Entrance	Measures the receptiveness of the entrance for persons with disabilities, assessing infrastructure preparedness such as the presence of ramps, disability-friendly infrastructure, slip resistance, and accessible counter heights.
Services	Evaluates the readiness of services offered concerning convenience, available space, reachability, and the use of assistive technology.
Toilet rooms	Focuses on the convenience of toilet rooms and the suitability of supporting equipment for persons with disabilities, including appropriate equipment height and width.
Waiting rooms	Assesses the ease of finding the path to the waiting room and its suitability for patients with disabilities, considering the availability of directional signs and ample space for wheelchairs.
Clinic rooms	Evaluates the accessibility of clinic rooms for persons with disabilities, examining clear pathways to clinic rooms and ensuring sufficient space within doorways and the rooms themselves.
Equipment	Assesses the availability of proper equipment to facilitate the treatment of persons with disabilities, including accessible examination tables and radiological equipment.
Process and training	Investigates the training and competence of healthcare providers regarding disability needs, including communication and guidance for patients with disabilities, as well as processes for identifying, notifying, and assisting persons with disabilities.

### Data analysis

Descriptive data analysis was conducted using SPSS V22.0, and the results were presented using percentages and frequencies. As the data did not follow a normal distribution, the Mann–Whitney U test was used to identify differences in domain scores between government-built buildings and rented houses.

## RESULTS

A total of 56 PHCs were assessed during three weeks. The assessment was carried out in person by eight surveyors using an electronic version of the survey. Of the 56 PHCs, 28 were government-built buildings, while the other 28 were rented houses. Categorized by the network, 10 PHCs belonged to the AlKhobar Network (18%), 20 PHCs belonged to the Dammam Network (36%), and 26 PHCs to the AlQatif Network (46%).

Table 2 provides an overview of the average accessibility scores, demonstrating that government-built buildings had an average accessibility score of 83.2%, while rented houses had a slightly lower average score of 67.1%. Moreover, the analysis across different networks revealed that Dammam had an average accessibility score of 77.9%, AlKhobar recorded an average of 78.4%, and AlQatif had an average accessibility score of 71.96% (Table 3).

**Table 2:** Overall scores by network and type of building

Average score by network	
Dammam	77.9
AlKhobar	78.4
AlQatif	71.9
**Average scores by type of building**	
Government-built buildings	83.2
Rented houses	67.1

**Table 3:** Average domain scores by type of building

Domain score by type of building		
Domain	Government-built buildings	Rented houses
Parking	83%	77%
Entrance	87%	72%
Services	75%	47%
Restrooms	70%	57%
Waiting rooms	79%	63%
Clinic rooms	93%	87%
Equipment	55%	56%
Process and training	42%	43%

**Figure 1:**
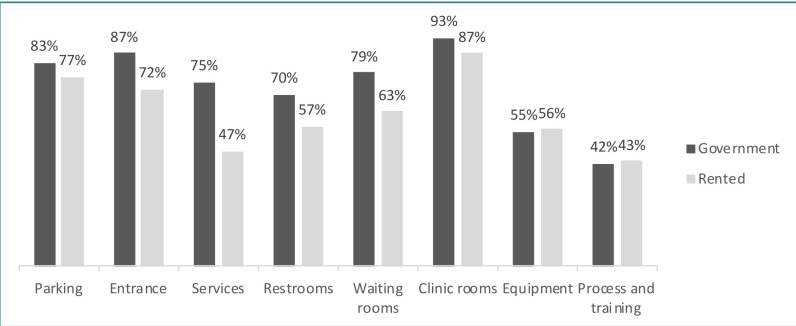
Building accessibility by domain type

Figure 1 shows the difference in scores between government-built buildings and rented houses. The most noteworthy differences were viewed in the services domain, where accessibility levels averaged 47% in rented houses compared to 75% in government-built buildings. Further analysis of the services domain showed that none of the rented houses had an elevator, which impacted their services domain accessibility. On the other hand, in government-built buildings, 38% of the facilities had an elevator. The overall level of the accessible parking domain was 80%, with 83% and 77% for government-built buildings and rented houses, respectively. However, 37% of the facilities assessed in this domain had sufficient space on at least one side of the parking space for passengers to exit the vehicle. Similar scores were found in the accessible entrance domain, with an average of 79%, 87% for government-built buildings, and 72% for rented houses. The accessible restrooms domain overall average was 64%, with 70% for government-built buildings and 57% for rented houses. In both building types, only 54% of facilities had restroom doors that were wheelchair accessible. The waiting rooms domain average was 71%, with 79% for government-built buildings and 63% for rented houses. In both facility types, 61% had waiting rooms that were easily identifiable with signs. The clinic rooms domain average for both building types was 90%; clinics in both building types had doorways that fit a wheelchair, and persons who used wheelchairs had enough space to move around comfortably (93% in governmental buildings and 87% in rented ones). The accessible equipment domain average was 55%. Only 38% of the facilities had a wheelchair-accessible weight scale, and less than 54% of facilities in both building types had accessible examination tables and chairs. The process and training domain accessibility level was less than 45% in both building types. Only 14% of the facilities had a designated healthcare provider to guide persons with disabilities within the facility. In addition, in both facility types, only 21% had an internal process for identifying persons with disabilities and assisting them. On average, 27% of the facilities trained healthcare providers on how to support persons with disabilities. The Mann–Whitney U test revealed that the differences in the domains between government-built buildings and rented houses were not statistically significant except for two domains. In the services domain, the results showed a significant difference with a P value of <.001. In addition, the entrance domain also has a statistically significant difference between both building types (P=.012).

## DISCUSSION

The growing global awareness of the importance of disability inclusiveness has led to an increasing number of studies examining the compliance of the built environment with accessibility legislation aimed at ensuring access for all individuals, including those with disabilities [13]. In the Kingdom of Saudi Arabia, the disability code specifies regulations in architecture to ensure accessibility for persons with disabilities. These regulations establish essential criteria that all educational institutions, medical facilities, habilitation centers, and public spaces must adhere to in their building designs to effectively accommodate persons with disabilities [4].

Although the Saudi Supreme Council is currently working with local relevant authorities to mandate adherence to the Disability Code, much work remains. The findings reported in the current study emphasize the potential difficulty experienced by persons with disabilities when visiting healthcare facilities. Our study found that government-built buildings had better overall accessibility than rented houses (83.2% and 67.1%, respectively).

Government-built buildings had significantly higher accessibility in the services domain than rented houses (P<.001). This was due to several factors, the most important being the lack of elevators in rented houses. In the facilities where elevators were not available, healthcare providers with clinics above the ground floor had to accommodate persons with disabilities by offering medical services on the ground floor. These separate accommodations could result in extended wait times as well as unexpected scheduling difficulties and clinic room availability. Such circumstances may lead to dissatisfaction with the services offered, potentially discouraging persons with disabilities from seeking healthcare services.

The comparison between government-built buildings and rented houses in the entrance domain showed a statistically significant difference in accessibility (P=.012). One of the factors that may have impacted the accessibility score in rented houses is having high counters in the reception area, which can lead to difficulties in communication between a person who uses a wheelchair and the receptionist. This may hinder communication and can lead to feelings of frustration and discouragement. Similar findings were reported by a study investigating architectural and transportation barriers to the accessibility of medical facilities for persons with disabilities in Peru [14]. The authors administered the Peruvian Disability survey to more than twenty thousand participants to measure the association between barriers and accessibility of persons with disabilities to healthcare facilities and rehabilitation centers. Consistent with this study, the authors found that the physical and environmental barriers faced by persons with disabilities affected their usage of healthcare centers.

In our findings, the clinic rooms domain received high scores in both types of buildings, with an average score of 90%. This means that persons with disabilities visiting most of the surveyed buildings had clear pathways to clinic rooms, ample space to navigate doorways, move within clinics, and access the entrance/exit of PHC. Similarly, a study assessing the accessibility of primary healthcare centers for persons with disabilities in India found that most accessible centers had ramps and clear pathways [15]. We believe that since PHCs are the first point of contact for those seeking governmentally provided non-urgent healthcare, simply entering the building is essential for receiving health services and is a step towards providing inclusive care for persons with disabilities.

Equipment specifically designed to support the needs of people with disabilities, such as wheelchair-accessible scales and disability-friendly examination beds, were absent in both building types. We make the case that the absence of proper equipment, without a doubt, impacts the services provided by healthcare staff to those presenting particularly with physical disabilities. For example, the inability to properly weigh a person with a disability may lead to improper vital sign documentation, triaging, and dosage, affecting the overall quality of care. Similarly, Nischith *et al*. [15] also noted that PHCs in their study lacked height-adjustable examination tables, wheelchair-friendly scales, and disability toilets, which hindered the care provided to persons with disabilities.

Although disability training for healthcare providers has been proven to increase the standard of care for persons with disabilities, generally, healthcare staff training on how to support patients with disabilities is inadequate [16]. We observed a similar trend throughout the facilities surveyed within the Eastern Region for both building types, where the process and training domain accessibility level was less than 45%. Some studies found that those who completed medical education were more likely to have negative attitudes toward disability than those who did not [16]. This emphasizes the importance of supplementing disability training, specifically for those who are working in primary care and are likely to encounter persons with disabilities. The lack of disability training for healthcare providers may lead to stigma, negativity, and ultimately to barriers to receiving quality medical care [17].

Our findings align with other studies regarding disparities in accessing governmental healthcare facilities between patients with and without disabilities [18, 19]. Interestingly, these studies attributed the variation in accessibility to differences in socioeconomic status rather than only the presence of a disability. It was explained that individuals with disabilities often face lower socio-economic circumstances, which can adversely affect their ability to access healthcare services, as supported by existing evidence [18, 19]. While our study offers a unique assessment of the infrastructural barriers in Eastern Saudi Arabia facing persons with disabilities, there is still limited understanding of the situation at hand. There is a need for further studies in Eastern Saudi Arabia looking into factors affecting the accessibility of persons with disabilities. These factors might include understanding and considering socioeconomic and educational status in communities to distribute resources and training better.

Given that persons with disabilities are frequent visitors to healthcare facilities [14], it can be argued that the absence of elevators, ramps, unaccommodating counters, and the limited training of healthcare providers in catering to the needs of persons with disabilities are significant contributors to disability-related barriers.

Recently, Saudi Arabia established the National Register of Disability and Persons with Disabilities Survey [20]. This registry could play a major role in contributing to a comprehensive needs assessment that supports facility planning, training, enacting, and implementing policies and ultimately supports the case for increasing the investment into government-built PHCs throughout the country. It is also necessary to encourage compliance with accrediting bodies. Through these means, PHCs could be persuaded to incorporate disability inclusion guidelines to reduce disparities and move towards nationwide accessibility and equitable care for all.

Internationally, the inclusion of persons with disabilities has been a concern that has brought forward many solutions. A study done in the United States advocated for the use of data to support healthcare decisions regarding persons with disabilities. Additionally, the study urges the prioritization of the inclusion of persons with disabilities in public health programs as well as training to support those with additional needs [21].

Recommendations provided by other health systems include using checklists for healthcare facilities to monitor adherence, guide the inclusiveness of persons with disabilities, and ultimately reduce disparities [14, 22].

## CONCLUSION

The present study sheds some light on the physical and infrastructural barriers faced by persons with disabilities in Eastern Saudi Arabia. Specifically, it indicates that persons with disabilities are more likely to face difficulties in accessibility when they are treated in a rented PHC. Although government-built buildings are more likely to have better accessibility, this study underscores that accessibility remains a significant challenge for persons with disabilities. Finally, the evidence from this study indicates that there is still a long way to go for government-built buildings to become disability-friendly. Nevertheless, they are better equipped and more accessible for persons with disabilities compared to rented PHCs. Therefore, it is recommended that future investments should prioritize governmentally owned facilities that adhere to disability codes. This would not only promote the utilization of healthcare services but also contribute to the overall health and well-being of persons with disabilities.
